# Postpartum Depression and Its Determinants: A Cross-Sectional Study

**DOI:** 10.7759/cureus.74044

**Published:** 2024-11-19

**Authors:** Puja Kumari, Saurav Basu

**Affiliations:** 1 Epidemiology and Public Health, Indian Institute of Public Health-Delhi, Delhi, IND; 2 Community Medicine, Employees' State Insurance Corporation (ESIC) Medical College and Hospital, Kolkata, IND

**Keywords:** depression, health systems, india, postpartum depression, women's mental health

## Abstract

Background

Postpartum depression (PPD) is the most prevalent psychological disorder after childbirth associated with a negative impact on the daily functioning of mothers and the cognitive development of infants. Inequitable primary mental health access in lower-middle-income countries (LMICs) further aggravates this major public health problem.

Objective

The objective of this study is to estimate the prevalence and determinants of PPD among women reporting to secondary care facilities in Delhi, India.

Methods

A cross-sectional study was conducted in the outpatient settings of two secondary care (one government and one private) hospitals in the Delhi-National Capital Region among mothers of infants aged below six months. The study was conducted from January to June 2023. Data were collected by a single trained investigator using a self-designed interview schedule, while PPD was measured by administering the Edinburgh Postnatal Depression Scale (EPDS). Data were analyzed with Stata statistical software, version 15.1 (StataCorp LLC, College Station, TX). P < 0.05 was considered statistically significant.

Results

The mean age of the study participants was 28.49 ± 3.77 years with 53 (18.28%) detected as having possible postpartum depression on screening with the EPDS. On adjustment for covariates, the participants with husbands consuming alcohol (adjusted odds ratio, 6.97; 95% confidence interval {CI}, 2.73-17.8), those who underwent C-section (adjusted odds ratio, 4.39; 95% CI, 1.02-18.85), and those giving birth in private hospitals (adjusted odds ratio, 5.48; 95% CI, 1.53-19.55) had significantly higher odds of having PPD. In contrast, mothers staying at home (not employed) (adjusted odds ratio, 0.08; 95% CI, 0.02-0.41), without specific preference for the newborn's gender (adjusted odds ratio, 0.07; 95% CI, 0.01-0.78), and those living in nuclear families (adjusted odds ratio, 0.03; 95% CI, 0.005-0.19) had significantly lower odds of PPD.

Conclusion

Nearly one in five mothers were screened for having possible PPD. Prioritizing birth preparedness during the antenatal period and strengthening health system screening protocols may prevent and mitigate the effects of PPD.

## Introduction

Depression is the leading cause of disease-related disability among women worldwide with major depression twice as prevalent in women compared to men [1]. Maternal deaths due to suicide, especially among women of childbearing age, is a public health concern, especially in lower-middle-income countries (LMICs) lacking effective mental health services with the coexistence of the social stigma of mental illness impeding their utilization [2].

Postpartum depression (PPD) is the most prevalent psychological disorder after childbirth, usually within the first year, and it may be harmful to the social and cognitive development of newborns and under-five children [3]. The first six months following delivery are crucial for the physical and mental development of a child [4]. PPD causes sadness, anxiety, and exhaustion and hinders caregiving and work abilities among new mothers [5,6]. Children of mothers with depression are likely to experience unreliable affection and impaired psychomotor and cognitive development [7,8]. Furthermore, PPD may impede mother-infant bonding and interaction, as well as the child's cognitive and emotional growth, thereby stunting the child's growth, especially in terms of intelligence and language, while mothers can be less attentive to their child's needs [9-13]. Young infants are especially susceptible to the unresponsive or rejecting care associated with PPD because of their reliance on mothers during the crucial imprinting period of infancy that engineers their neuropsychological development [14].

Optimized mental health service availability is extremely limited in most LMICs with disparities in their access due to inequitable health systems. Major depression rates vary by location, occurring three times more frequently in LMICs compared to richer countries, and thereby, major depression constitutes a major global public health concern that adversely impacts women and their families [15,16]. A systematic review of 47 studies from 18 LMICs estimated a PPD prevalence of 18.6% (95% confidence interval {CI}: 18.0-19.2) [15]. However, another meta-analysis, including 143 studies from 40 countries, observed the PPD prevalence rate to vary from 0.5% to nearly 60% [17]. In contrast, during the first year following birth, postpartum depression prevalence in Western countries ranges from 10% to 15% [18].

A meta-analysis of studies from India, the country with the largest birth cohort of nearly 27 million newborns, estimated the pooled prevalence of postpartum depression as 22% [19]. Another multicountry study reported that the proportion of women with mental distress 6-18 months following childbirth was 30.5% indicating the high magnitude of this important public mental health problem [20]. Nevertheless, despite the substantial reduction in maternal mortality in India since 2014, a lack of programmatic focus on PPD is conspicuous by the absence of existing reproductive and child health programs [21]. Hence, the early detection and prompt management of postpartum depression warrant high prioritization for attaining positive maternal and child health outcomes.

Most Indian studies have assessed PPD in government health settings, but there is a lack of recent comparative assessment from private health settings. Moreover, few studies have been recently conducted in Northern India, and none have obtained perspectives on PPD from medical and nursing practitioners [19,22]. Therefore, we conducted this study with the primary objective of determining the prevalence and predictors of PPD among women reporting to secondary care facilities in Delhi, India. As a secondary objective, we also assessed perspectives on PPD management from doctors and nurses working in the same health settings. The findings will aid decision-makers in the planning and prioritization of mental health services to address PPD among Indian women, as well as in understanding the challenges and opportunities for health system strengthening necessary for effective PPD prevention and management.

## Materials and methods

Design, setting, and participants

A cross-sectional study was conducted at a secondary care private hospital and a secondary care government hospital, signifying a level of care within the Indian health system, which is consistent with the availability of some specialist care, especially the availability of emergency services including caesarean section (CS). Convenience sampling was done for selecting the hospitals in the Delhi-National Capital Region. Data collection was conducted from January to June 2023. Mothers between the ages of 18 and 39 who had given birth within the past six months were included in the study. In order to capture mothers with likely persistent PPD and mitigate recall bias, the inclusion criterion was mothers who underwent childbirth within the past six months. Factors that were likely confounders and suggestive of pre-existing psychiatric illness or depression were excluded from the study including mothers with a history of intake of antidepressant medication and those with a history of newborns with congenital abnormalities or mortality [23]. Women with a history of infertility were also excluded as it is a well-established risk factor for PPD [24,25]. The participants were recruited from mothers attending the outpatient departments of either gynecology, neonate/pediatric, or immunization clinic at the selected hospitals.

Sample size and sampling

The sample size for this study was estimated considering 95% confidence intervals, 20% expected prevalence of PPD using a pooled estimate from India, and 5% absolute precision and also accounting for a 10% non-response rate [19]. The final sample size was calculated to be 290 with 145 participants sampled from both the hospitals. Consecutive sampling was used for the selection of the study participants within the health facilities.

Methodology

Data were collected by a trained single investigator using a self-designed interview schedule, which collected information on personal history, occupational history, family type, obstetric history, and other risk factors. PPD was screened using the English and Hindi versions of the Edinburgh Postnatal Depression Scale (EPDS), which is validated in Indian settings for identifying probable depression among antenatal and postpartum women [25-27]. The EPDS consists of 10 items graded on a four-point scale, with a total score range of 0-30. According to the severity or duration of each symptom, each question is graded on a four-point scale from 0 to 3, and it takes about 10 minutes to complete. An EPDS score of above 13 indicates the presence of moderate to severe depressive symptoms [28-30].

In this study, an EPDS score of ≥13 was considered as "possible depression," while a score of <13 was interpreted as "no depression" by adopting the screening guideline recommended by the Centre of Perinatal Excellence, Australia [31]. A relatively higher cutoff was selected for considering the likely presence of PPD among the participants, which may have not only reduced the overall sensitivity of the tool but also reduced the false positivity rate, which is necessary considering the limited resources for managing PPD in resource-limited settings.

The EPDS alone does not provide a diagnosis of depression, which requires evaluation by qualified healthcare professionals. Furthermore, the scale is ineffective at identifying mothers who have pre-existing neurosis, phobias, or personality disorders [29,30]. The questionnaire was administered in Hindi or English, depending on the participant's reading and writing proficiency in the respective languages, and also verbally administered in the language of their choice upon request.

The socioeconomic status (SES) of the participants was ascertained using the BG Prasad scale, which is validated in both rural and urban populations of India. The per capita monthly income of the household that was adjusted for inflation with the Consumer Price Index for Industrial Workers in India for July 2022 is used in the scale to stratify SES into five categories from class I to class V with the former signifying the highest SES and the latter signifying the lowest SES [32].

To assess the perspectives of healthcare providers on PPD, a total of four doctors and four nurses from the gynecology and obstetrics departments of the hospitals were interviewed using six open-ended questions on PPD significance, screening, counselling methods, prevention approaches, programmatic inclusion, and strategies to prevent postpartum depression.

The final study tool was used to create an Excel database with built-in checks. Paper forms were used to collect data in the field, which was then entered into the database. Electronic data were stored in password-protected files. Hard copies of the interview and consent forms were securely kept by the principal investigator, who maintained confidentiality. Codes were used to link the data to the original information.

Statistical analysis

Data were analyzed with Stata statistical software, version 15.1 (StataCorp LLC, College Station, TX). Quantitative variables were expressed as mean and standard deviation, whereas categorical variables were expressed as frequency and proportions. The chi-square test was used to establish an association between the categorical variables. To find out the determinants of postpartum depression, a binary logistic regression was conducted by taking PPD as the outcome variable and including statistically significant independent variables from bivariate analysis as covariates. P < 0.05 was considered statistically significant. The interviews with healthcare providers fit into a priori themes, which were subsequently summarized.

Ethical considerations

Approval for the study was obtained from the Institutional Ethics Committee of the Indian Institute of Public Health-Delhi; vide IIPHD_IEC_MPH_S_28_2023 dated 17.01.2023. Administrative approval for conducting the study was obtained from the health facilities. Written and informed consent was obtained from all the participants. Interviews with participant mothers were conducted in a separate room to ensure confidentiality. The women screened with probable PPD were counselled on the possible diagnosis and instructed to meet their gynecologist for further assessment.

## Results

We enrolled a total of 290 married women with infants aged less than six months. The response rate of the survey was 100%, with none of the eligible women declining participation in the study. The mean age of the study participants was 28.49 ± 3.77 years. The majority of mothers were housewives (52.41%), living in joint families (56.55%), and belonging to the upper socioeconomic class (52.76%), while more than two in three had a higher secondary education or above (71.72%) (Table 1).

**Table 1:** Distribution of sociodemographic and obstetric factors among the study participants (N = 290)

Variables	Categories	Frequency (n) (%)
Education	Up to primary	22 (7.59)
	Secondary	60 (20.69)
	Higher secondary and above	208 (71.72)
Religion	Hindu	197 (67.93)
	Non-Hindu (Muslim and others)	93 (32.07)
Husband tobacco consumption	Yes	36 (12.41)
	No	254 (87.59)
Husband alcohol consumption	Yes	87 (30)
	No	203 (70)
Present employment status	Employed	125 (43.1)
	Self-employed	13 (4.48)
	Unemployed	152 (52.41)
Family type	Joint	164 (56.55)
	Nuclear	126 (43.45)
Socioeconomic class	I (8220 and above)	153 (52.76)
	II (4110-8219)	96 (33.10)
	III (2465-4109)	36 (12.41)
	IV (1230-2464)	5 (1.72)
Type of pregnancy	Planned pregnancy	143 (49.31)
	Unplanned pregnancy	147 (50.69)
Antenatal medical illness	Yes	28 (9.66)
	No	262 (90.34)
Complication during delivery	Yes	22 (7.59)
	No	262 (92.41)
Mode of last delivery	Vaginal	92 (31.72)
	C-section	198 (68.28)
Term status	Preterm	34 (11.72)
	Term	256 (88.28)
Total children	1	219 (75.52)
	2	66 (22.76)
	3	5 (1.72)
Male children	0	85 (29.31)
	1	203 (70)
	2	2 (0.69)
Female children	0	145 (50)
	1	131 (45.17)
	2	13 (4.48)
	3	1 (0.34)

The prevalence of PPD was 18.28% (n = 53; 95% CI: 14-23.2) considering those individuals with an EPDS score of ≥13. Furthermore, among women who delivered in government hospitals (n = 145), only 13 (~9%) were identified with PPD based on their EPDS scores, while in contrast, among women who delivered in private hospitals (n = 145), 40 (27.6%) were detected with PPD (p < 0.001).

The bivariate analysis revealed significant associations between PPD and the following factors: educational status, the type of delivery, present employment status, and family type. Working mothers with higher secondary education who had undergone a caesarean section and belonged to the upper socioeconomic class of joint families had a higher prevalence of PPD. However, although a significant number of first-time mothers experienced depressive symptoms, the relationships were not statistically significant (Table 2).

**Table 2:** Bivariate analysis of sociodemographic and obstetric factors associated with postpartum depression EPDS: Edinburgh Postnatal Depression Scale

Characteristic	Categories	Probable depression (EPDS ≥ 13), n (%)	Without depression (EPDS ≤ 12), n (%)	P-value
Age (years)	18-24	3 (5.66)	30 (12.66)	0.390
	25-32	43 (81.13)	177 (74.68)	
	33-39	7 (13.21)	30 (12.66)	
Education	Up to secondary	7 (13.21)	75 (31.65)	0.007
	Higher	46 (86.79)	162 (68.35)	
Religion	Hindu	48 (90.57)	149 (62.87)	<0.001
	Non-Hindu	5 (9.43)	88 (37.13)	
Husband tobacco consumption	Yes	12 (22.64)	24 (10.13)	0.012
	No	41 (77.36)	213 (89.87)	
Husband alcohol consumption	Yes	36 (67.92)	51 (21.52)	<0.001
	No	17 (32.08)	186 (78.48)	
Present employment status	Employed	46 (86.79)	79 (33.33)	<0.001
	Self-employed	4 (7.55)	9 (3.80)	
	Staying at home (not employed)	3 (5.66)	149 (62.87)	
Family type	Joint	51 (96.23)	113 (47.68)	<0.001
	Nuclear	2 (3.77)	124 (52.32)	
Socioeconomic class	I	35 (66.04)	118 (49.79)	0.10
	II	13 (24.53)	83 (35.02)	
	III/IV	5 (9.43)	36 (15.19)	
Comorbidities	Yes	4 (7.55)	16 (6.75)	0.769
	No	49 (92.45)	221 (93.25)	
Unwanted gender of the baby	Yes	9 (16.98)	11 (4.64)	0.001
	No	44 (83.02)	226 (95.36)	
Type of pregnancy	Planned	38 (71.70)	105 (44.30)	<0.001
	Unplanned	15 (28.30)	132 (55.70)	
Antenatal medical illness	Yes	7 (13.21)	21 (8.86)	0.333
	No	46 (86.79)	216 (91.14)	
Complication during delivery	Yes	5 (9.43)	17 (7.17)	0.574
	No	48 (90.57)	220 (92.83)	
Mode of last delivery	Vaginal	4 (7.55)	88 (37.13)	<0.001
	C-section	49 (92.45)	149 (62.87)	
Week of last delivery	Preterm	7 (13.21)	27 (11.39)	0.710
	Term	46 (86.79)	210 (88.61)	
Total number of children	1 child	44 (83.02)	175 (73.84)	0.160
	≥2 children	9 (16.98)	62 (26.16)	
Number of male children	None	9 (16.98)	76 (32.07)	0.029
	≥1 child	44 (83.02)	161 (67.93)	
Number of female children	None	38 (71.70)	107 (45.15)	<0.001
	≥1 child	15 (28.30)	130 (54.85)	

The logistic regression analysis revealed that age, education, religion, socioeconomic status, the type of pregnancy (planned/unplanned), the number of male or female children, and a history of illness during the antenatal period in the mothers lacked any statistically significant association with the presence of PPD. Further, the mothers reporting alcohol consumption by their husbands (adjusted odds ratio, 6.97; 95% CI, 2.73-17.8), those who underwent C-section (adjusted odds ratio, 4.39; 95% CI, 1.02-18.85), and those giving birth in a private hospital (adjusted odds ratio, 5.48; 95% CI, 1.53-19.55) had significantly higher odds of having PPD. In contrast, mothers who were staying at home (not employed) (adjusted odds ratio, 0.08; 95% CI, 0.02-0.41), satisfied with the gender of the newborn (adjusted odds ratio, 0.07; 95% CI, 0.01-0.78), and lived in nuclear families (adjusted odds ratio, 0.03; 95% CI, 0.005-0.19) had significantly lower odds of PPD (Table 3).

**Table 3:** Logistic regression analyses of factors associated with postpartum depression Ref indicates the reference value CI: confidence interval

Variables	Categories	Crude odds ratio (95% CI)	Adjusted odds ratio (95% CI)
Age	18-24 years	Ref	-
	25-32 years	2.43 (0.71-8.3)	-
	33-39 years	2.33 (0.55-9.89)	-
Education	Up to secondary	Ref	Ref
	Higher	3.04 (1.31-7.05)	0.61 (0.12-3.11)
Religion	Hindu	Ref	Ref
	Non-Hindu	0.18 (0.07-0.46)	1.54 (0.28-8.49)
Husband alcohol consumption	No	Ref	Ref
	Yes	7.72 (4.01-14.86)	6.97 (2.73-17.8)
Present employment status	Employed	Ref	Ref
	Self-employed	0.76 (0.22-2.61)	3.14 (0.39-25.45)
	Unemployed	0.03 (0.01-0.11)	0.08 (0.02-0.41)
Family type	Joint	Ref	Ref
	Nuclear	0.04 (0.01-0.15)	0.03 (0.005-0.19)
Socioeconomic status	Upper class	Ref	-
	Upper middle class	0.53 (0.26-1.06)	-
	Middle class to lower middle class	0.47 (0.17-1.28)	-
Unwanted gender of the baby	Yes	Ref	Ref
	No	0.24 (0.09-0.61)	0.07 (0.01-0.78)
Type of pregnancy	Planned pregnancy	Ref	Ref
	Unplanned pregnancy	0.31 (0.16-0.60)	2.58 (0.75-8.9)
Mode of last delivery	Vaginal	Ref	Ref
	C-section	7.23 (2.52-20.73)	4.39 (1.02-18.85)
Number of male children	None	Ref	Ref
	≥1 child	2.31 (1.07-4.97)	5.56 (0.55-56.55)
Number of female children	None	Ref	Ref
	≥1 child	0.32 (0.17-0.62)	1.15 (0.27-4.98)
Health setting	Government hospital	Ref	Ref
	Private hospital	3.87 (1.97-7.61)	5.48 (1.53-19.55)
Antenatal medical illness	No	Ref	-
	Yes	1.57 (0.63-3.90)	-

Healthcare professionals perceived PPD as a serious problem but indicated that the lack of routine protocols, training, and hospital policies for PPD management within their health facilities undermined its effective resolution. They believed the screening, counselling, and early recognition of PPD needed strengthening of existing services through improved protocols, training programs, and formulating hospital policies in this regard (Table 4).

**Table 4:** Postpartum depression management perspectives among doctors and nurses

Theme	Description
Postpartum depression (PPD) is a serious problem	Participants perceive postpartum depression as a serious problem, emphasizing the need for screening, counselling, and addressing depressive symptoms for the well-being of both the mother and the infant. Postpartum depression can negatively affect a mother's ability to care for her newborn baby
Absence of routine protocol for PPD management	Participants agree that there is no existing routine protocol for postpartum depression in both hospitals, indicating a lack of standardized procedures for identifying and managing postpartum depression cases. Some suggest including simple depression assessments as part of routine postnatal checkups for early recognition and treatment
Lack of training for effective screening and management of women with PPD	All participants state that they have not received any training specifically focused on postpartum depression in both hospitals. They express a willingness and interest in receiving training for the better understanding and management of postpartum depression cases
Treatment and referral pathways for women with PPD	Participants mention that patients exhibiting postpartum depression symptoms often consult their gynecologists initially. If patients present with mood changes, anxiety, self-harm thoughts, or feeling miserable, they are referred to the psychiatric department for further evaluation and treatment
Absence of an existing hospital policy for PPD management	Participants indicate that there is no existing policy or protocol specifically addressing postpartum depression in both hospitals, suggesting a lack of formal guidelines or procedures for managing postpartum depression cases

## Discussion

Postpartum depression is a significant public health challenge in the developing world, but health system preparedness in the early detection and management of the condition is suboptimal. The present study observed the prevalence of PPD in 18.28% of mothers with infants below six months of age, which is comparatively lower than a meta-analysis estimate (22%) of 38 studies from India [19]. The burden of PPD was also lower in this study compared to another study (29%) conducted in a primary health facility in an urban slum and resettlement colony in Delhi among women having an infant child [22]. Variations in PPD burden estimates can be due to demographic heterogeneity since it is well-established that postpartum depression is usually more common in women of younger age, those having lower educational attainments, and those belonging to low socioeconomic status [16,23,33,34]. However, in this study, these variables were not independently associated with PPD in the adjusted model. Similarly, a large study from Japan observed the association of education status with PPD in the bivariate model but a weak relationship in the adjusted model [35]. A possible reason for the lack of association of PPD with educational status observed in the present study can be the high level of mental health stigma, poor health-seeking behavior, and the lack of awareness related to mental health problems that is significantly generalizable among women in India [36,37]. Similarly, women who were unemployed had a lower risk of PPD in this study, which contradicts the evidence from developed countries where employment is considered to have a possible protective effect against depression [38]. The type and patterns of employment of postpartum women can substantially differ in LMICs and high-income countries, with low-skilled and low-income work in the former possibly contributing to a higher risk of PPD when compared with women engaged in comparatively high-skilled and high-paying work.

In this study, PPD was more prevalent among women living in joint families despite the possibly higher family support within such multi-generation families living under one roof. Another study similarly observed a higher burden of PPD in Chinese women living with their parents-in-law [39]. These findings may be possibly attributed to disagreements and conflicts of young mothers with their in-laws and increased obligations and domestic responsibilities contributing to higher vulnerability.

In this study, mothers who had caesarean section (CS) deliveries had more than four times higher odds of having PPD, a finding which corroborates evidence from a global systematic review suggestive of the increased risk of PPD in the presence of CS, especially in the initial six months postpartum [40].

Women who reported their husbands consuming alcohol had nearly seven times higher odds of PPD compared to women whose husbands abstained from PPD. Husband alcoholism is frequently associated with intimate partner violence in low-income settings in LMICs, although the latter could not be ascertained in this study due to non-response among the participants [41].

Further, in the present study, women with a specific preference for the newborn's gender, which did not materialize, had significantly higher odds of PPD. In India, the cultural preference for male children has been historically well-established since antiquity with accentuation in the postcolonial period. Another study from a tertiary care hospital in Delhi observed a significantly higher prevalence of PPD among women who gave birth to female infants [42]. There is similar evidence that women in China who deliver a girl child have a higher risk of PPD, although the prevalent one-child policy in the country may also contribute to the phenomenon, a situation dissimilar to India [43].

The present study has certain public health implications. Mothers living in joint families and delivering their last child by C-section have higher odds of experiencing PPD suggestive of the need for prioritizing comfortable living arrangements, and birth preparedness, during the antenatal period. The training and sensitization of health professionals for screening with questionnaires, early detection, management, and referral of mothers with symptoms of depression require enhanced focus in Indian health settings. National health programs in India should incorporate strategies and interventions for the early recognition and effective management of PPD. The promotion of evidence-based behavioral modifications such as tobacco cessation and physical activity can also be integrated within essential health packages for the care of mothers with infants [44]. Expanding the role of telemedicine in the management of PPD and promoting the availability and dispensing of antidepressant medications through trained primary care providers also warrant exploration in low-resource settings [45].

The strengths of the study are that it included both a government and a private health facility, thereby increasing the representativeness of the sample and the external validity of the study results. Furthermore, the data were collected by a trained single investigator, reducing the chances of inter-observer bias.

However, there are also certain study limitations. The design lacked an assessment of temporal association that precluded causal inference. Women up to six months postpartum were included in this study to minimize recall bias, but previous evidence suggests that the burden of PPD is higher in the initial months following childbirth, thereby limiting the generalizability of the findings among women who were >6 months postpartum. The study sites were restricted to outpatients in secondary care hospitals; consequently, the study findings may have reduced external validity when considering tertiary care settings where complicated postpartum cases may have higher follow-up rates. In-patients were excluded from the present study as their current health status (illness) and likely financial burden from the cost of illness could have contributed to stress and anxiety and precluded the accurate evaluation of their mental health status. Further, interviewing women in the pediatric OPD may overestimate PPD due to associated child-related stressors. Community-based studies are needed for a more accurate estimation of PPD, as depressed and vulnerable women may have poor health-seeking behavior and tend to remain isolated at home. Finally, this study lacks qualitative insights from women detected with likely PPD on screening with EPDS, their coping mechanisms, and their treatment-seeking behaviors.

## Conclusions

Nearly one in five mothers of infants below six months of age were screened with possible PPD. Women reporting alcohol consumption by their husbands, caesarean section in a previous delivery, and giving birth in a private hospital had significantly higher odds, while women living in nuclear families, staying at home only (not employed), and satisfied with the gender of the newborn had significantly lower odds of PPD.
